# Supplementary material for: Reproducible acquisition, management and meta-analysis of nucleotide sequence (meta)data using q2-fondue
Source: Bioinformatics. 2022 Sep 20;38(22):5081–91. doi: 10.1093/bioinformatics/btac639 (PMC9665871; doi:10.1093/bioinformatics/btac639)
Supplement: btac639_Supplementary_Data [file btac639_supplementary_data.docx]

Supplementary Materials for

“Reproducible acquisition, management, and meta-analysis of nucleotide sequence (meta)data using q2-fondue”

Considering that fetching sequences is the most computation-intensive part of *q2-fondue*, we compared the run times of the *get-sequences* action to demonstrate the potential benefits of parallelization. As, following the download itself, the sequences need to be post-processed to ensure data integrity and consistent naming, we could see that increasing the number of parallel jobs from 1 to 4 significantly decreased the total run time of that action (Sup. Fig. 1).

| 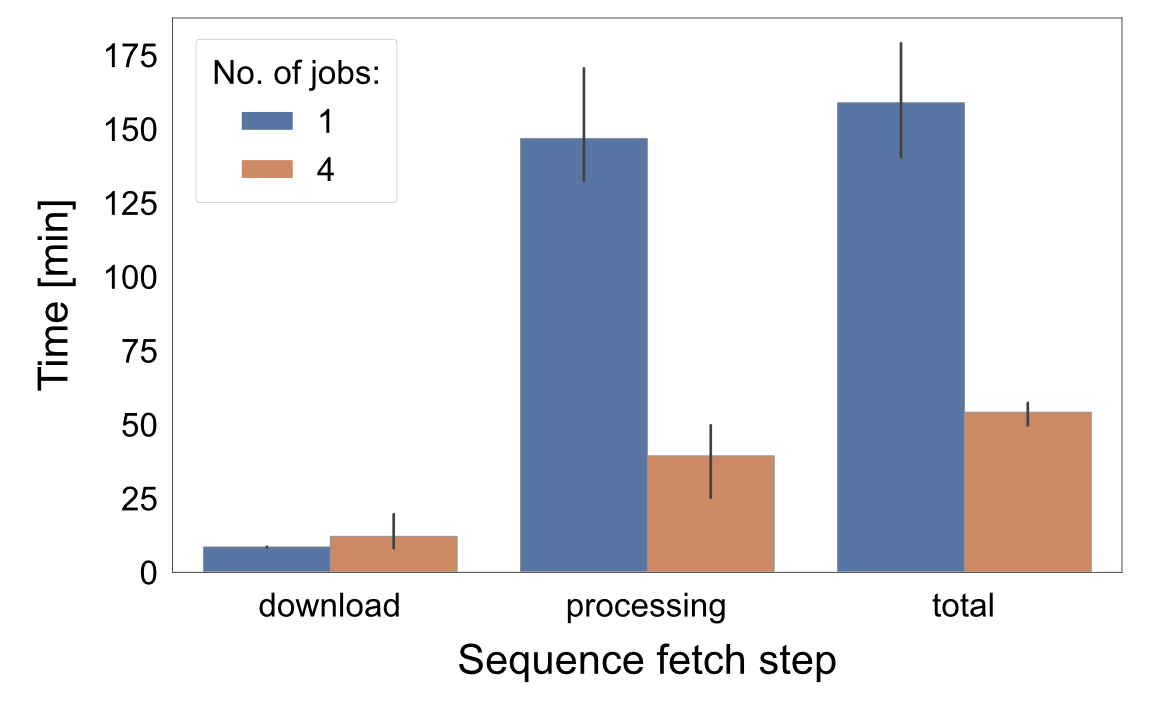 |
| --- |
| **Supplementary Figure 1.** Time to fetch and process sequences for 20 randomly selected run IDs using *get-sequences* action from *q2-fondue*. While the download phase is not affected by changing the number of jobs, the post-processing time (and, as a consequence, the total run time) is significantly shorter when using additional workers. Each data fetch iteration was repeated three times - bars represent an average time in minutes and the whiskers indicate the corresponding standard deviation. |
